# rBEFdata: documenting data exchange and analysis for a collaborative data management platform

**DOI:** 10.1002/ece3.1547

**Published:** 2015-07-03

**Authors:** Claas-Thido Pfaff, Birgitta König-Ries, Anne C Lang, Sophia Ratcliffe, Christian Wirth, Xingxing Man, Karin Nadrowski

**Affiliations:** 1 Department of Special Botany and Functional Biodiversity, University of LeipzigJohannisallee 21, 04103, Leipzig, Germany; 2 Department of Mathematics and Computer Science, Friedrich Schiller University of JenaErnst-Abbe-Platz 2, 07743, Jena, Germany

**Keywords:** Biodiversity ecosystem functioning, data postprocessing, data sharing, ecological metadata language, metadata, open science, open source, paper proposals, R, reproducible science, semantic integration, workflow

## Abstract

We are witnessing a growing gap separating primary research data from derived data products presented as knowledge in publications. Although journals today more often require the underlying data products used to derive the results as a prerequisite for a publication, the important link to the primary data is lost. However, documenting the postprocessing steps of data linking, the primary data with derived data products has the potential to increase the accuracy and the reproducibility of scientific findings significantly. Here, we introduce the rBEFdata R package as companion to the collaborative data management platform BEFdata. The R package provides programmatic access to features of the platform. It allows to search for data and integrates the search with external thesauri to improve the data discovery. It allows to download and import data and metadata into R for analysis. A batched download is available as well which works along a paper proposal mechanism implemented by BEFdata. This feature of BEFdata allows to group primary data and metadata and streamlines discussions and collaborations revolving around a certain research idea. The upload functionality of the R package in combination with the paper proposal mechanism of the portal allows to attach derived data products and scripts directly from R, thus addressing major aspects of documenting data postprocessing. We present the core features of the rBEFdata R package along an ecological analysis example and further discuss the potential of postprocessing documentation for data, linking primary data with derived data products and knowledge.

## Introduction

Large amounts of ecological data are gathered each year by researchers worldwide, striving to enhance the knowledge on our ecosystems. Many data management platforms and tools have been developed with the growing awareness on the value of data, the need of data curation, and the potential of data reuse (BEFdata, Nadrowski et al. 2013; Bexis, Lotz et al. 2012; Metacat, Berkley et al. 2001; DataOne, KNB). Data platforms and networks facilitate the access to heterogeneous data typically generated by ecological research projects. This is reflected in the variety of study systems, methods, data types, environmental contexts, and the temporal and spatial scales. A specific reuse of data is the fusion of many datasets in meta-analyses. This is of particular interest in ecology as it potentially allows quantitative summaries of research domains to generate higher-order conclusions about general trends and patterns (Arnqvist & Wooster 1995, Koricheva et al. 2013). Conclusions derived from analyzing data are then archived as papers in journals. Although an increasing number of journals today require the data used to derive the results as prerequisite for publication (e.g., f1000), the steps on how these data have been assembled from primary data and how the data have been processed during the analysis are often hidden. Losing the link between primary data, derived data products, and knowledge results in a “gulf” between primary data repositories and knowledge repositories (Shotton 2009; Attwood et al. 2010).

Linking primary data with a research idea in a proposal is a sociocultural mechanism applied by an increasing number of research consortia (e.g., Stokstad 2011). While this potentially helps to prevent duplication and to maximize synergies in projects, it lacks a persistent and structured documentation. The BEFdata (Biodiversity and Ecosystem Functioning Data) platform provides an implementation of this mechanism as a step-by-step wizard guiding through the creation of a proposal (Nadrowski et al. 2013). This mechanism does not only streamline the discussion and collaborations in research projects but also serves for documentation and thus as a single point of reference for information revolving around a research idea. It has the potential to link primary data, the research idea in the form of a rationale, and the derived data products in one place. Documenting the postprocessing steps of primary data towards a final data product has the potential to reduce redundant efforts in projects and increase the accuracy and the reproducibility of scientific findings. However, we need an easy way to add information to a paper proposal right at where the data are used, for example, in statistical scripting environments. We here present the core features of the r-Biodiversity and Ecosystem Functioning Data R package (rBEFdata) along an ecological analysis example highlighting the use of the proposal mechanism for documentation purposes. We further discuss the potential of data postprocessing documentation linking primary data with derived data products and knowledge to help building a bridge over the “gulf” between primary data and knowledge repositories.

## The BEFdata Portal

BEFdata is a data management web application written in Ruby on Rails (Nadrowski et al. 2013). It is open source software released under an MIT license. The development of the platform is driven by the joint Chinese–German–Swiss research project “BEF-China” (FOR 891). The source code as well as information on how to set up the application is provided via the Github repository and the associated wiki pages (https://github.com/befdata/befdata). The BEFdata portal is not comparable to large data archives (e.g., Gbif, DataOne) but is instead meant to be used by research collaborations during a period in which the data are not yet ready for being deposited in large international archives. The portal gives researchers in a project the opportunity to centrally store, clean, harmonize, and share their data in a private and secure environment. It uses the Ecological Metadata Language standard (EML, Fegraus et al. 2005) to format metadata, and it facilitates collaboration using a paper proposal mechanism (Nadrowski et al. 2013). Data hosted on instances of BEFdata are private by default, but the metadata is readable by everybody. A researcher can fill a shopping cart with data needed to answer certain research questions. After the selection of the data, a proposal has to be written to inform the data owners. This represents a mechanism that goes far beyond a simple data request by providing a transparent way of communicating the scope and rationale of a planned analysis to all researchers, listing the datasets required for the analysis as well as the potential co-authors. The analysis can start after all participants involved in the proposal agreed on a modus to work on toward a publication. For a more detailed description about the portal functionality, we refer to Nadrowski et al. 2013.

## Data for the Examples

**Figure 1:**
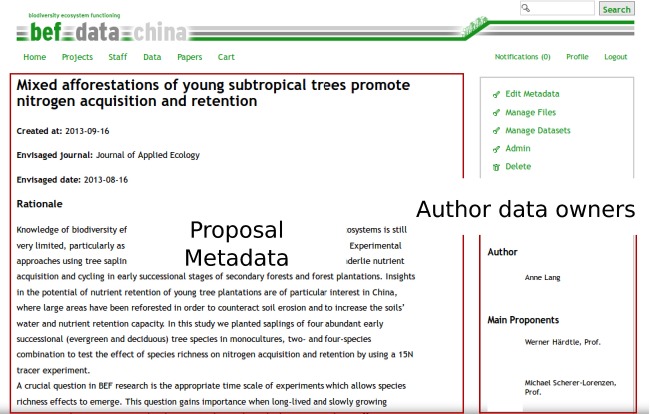
The page of the proposal we used in our example in this paper. Proposals serve as starting point for analyzing data hosted on BEFdata platforms. They can serve as central point of coordination and for documentation. They aggregate information such as the title, the author, and the date of creation as well as the envisaged journal and a detailed rationale. After an analysis, derived data products and scripts can be attached to complement this information. The proposal page that is shown here is available under the url http://china.befdata.biow.uni-leipzig.de/paperproposals/90 and the analysis has been published by Lang et al. 2014.

The BEFdata portal is currently used by two research consortia, the BEF-China (Bruelheide et al. 2014) and the FunDivEUROPE project (http://www.fundiveurope.eu). For our examples, we use data provided by the BEF-China project consortium. It is an international research collaboration with the aim to disentangle the role of tree and shrub diversity for production, erosion control, element cycling, and species conservation in Chinese subtropical forest ecosystems (Bruelheide et al. 2014). The research data of the BEF-China experiment are hosted on a BEFdata server available via http://china.befdata.biow.uni-leipzig.de. The data for our example analysis have been compiled in a paper proposal revolving around questions about nitrogen acquisition and retention in the study plots of the BEF-China experiment (Lang et al. 2014). The data have been collected by different subprojects of the BEF-China experiment and are publicly available via the paper proposal depicted in Figure1. The vocabulary used in the data discovery example is a work in progress thesaurus created for the domain of Biodiversity and Ecosystem Functioning. It is developed in the context of the project called the German Federation for Biological data (GFBio) and can be accessed via http://tematres.befdata.biow.uni-leipzig.de/vocab/.

## The rBEFdata Package

The package is developed for the R statistical programming environment (R Development Core Team 2015), and is part of the rOpenSci (http://ropensci.org/) package portfolio. It is released under an MIT license and a stable version is available via CRAN. We here introduce the rBEFdata R package and its core features available with version 0.3.5. rBEFdata provides programmatic access to features of the BEFdata data management platform. It allows to search for data and integrates with external thesauri hosted via a TemaTres vocabulary server (http://www.vocabularyserver.com) to improve the data discovery. rBEFdata allows to download and import data and metadata into R hosted on a BEFdata instance. A batched download is available as well which works along the paper proposal mechanism of BEFdata. This fetches all data associated with a specific research idea in one single step for analysis. Upload functionality of the R package allows to push new data to the BEFdata portal and to attach derived data products right from within R to their original paper proposal.

rBEFdata has a set of options that, for example, determine which instance of BEFdata is used. Issuing the command *bef.options()* provides an overview about the options available and their current values (in the following, all R commands embedded into the text appear in italics). Most of the option fields come with predefined values (related to BEF-China). This can be simply changed by the assignment of a new value. For example, the URL to an own BEFdata server can be set by *bef.options(*“*url*” = “http://my.own.befdata.instance.com”). This allows one to use the R package with one or several different instances of BEFdata. Other options are dedicated to the URL of a TemaTres vocabulary server and the name of a download folder. This folder is created to store downloads, for example, attachments of paper proposals like R scripts or images. For a detailed overview about all available commands, we refer to the package manual, which is available here http://cran.r-project.org/web/packages/rbefdata/rbefdata.pdf.

## Data Discovery and Thesaurus Integration

Datasets can be tagged with keywords on BEFdata portals. These keywords can then be used for data discovery from R issuing the command *bef.portal.get.dataset.for_keyword(“carbon”)*. Here in this example, we search for all data tagged with “carbon”. Multiple keywords can be used in combination with concatenation, for example c(“carbon”, “nitrogen”). The command returns a data frame with the titles and the IDs of the data found. The IDs can then be used to access the data with bef.portal.get.dataset_by(id = xx). In a second step, we improve the search along an external thesaurus using the TemaTres server integration. If we search the BEF-China instance of BEFdata for “plant organ” and “weight” with *bef.portal.get.dataset.for_keyword(c(“plant organ”, “weight”)*, which returns 24 datasets, this misses data that are tagged with narrower terms for plant organs. We access the external thesaurus to improve the search terms used towards more detailed terms. We receive a list of narrower terms issuing the command *bef.tematres(task = “fetchDown”, term = “plant organ”)*. The list returned from the vocabulary server then includes “leaf”, “root”, “twig” “seed” and “stem”. Repeating the search from the above with the extended list not only yields more-than-twice the number of datasets, but also results in the data tagged with the narrower terms (57). Exchanging the external thesaurus via the options command of rBEFdata allows to use an own or reuse existing vocabularies of preference like e.g. the LTER thesaurus.

## Data Access

**Figure 2:**
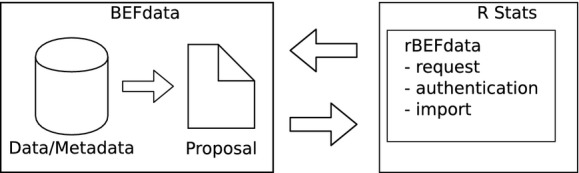
In our example workflow with rBEFdata, we first download and import all data that have been attached to the proposal depicted in Fig.1. You can check out the detailed R code via a Gist we made available on GitHub (https://gist.github.com/cpfaff/2a927c772342fe398466). Furthermore, the script containing all code of our examples in one file is available via figshare http://dx.doi.org/10.6084/m9.figshare.1365364 and Gist from here https://gist.github.com/cpfaff/c7dcfa1c971ee61150b2.

**Figure 3:**
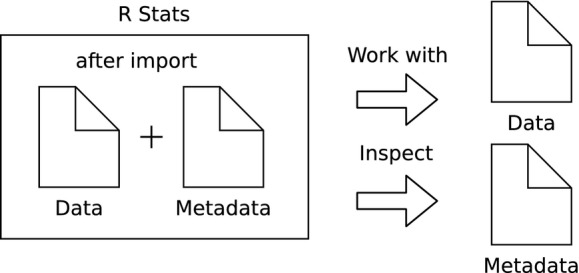
After the import of the data from the BEFdata platform, we can start working with it right away. Furthermore, we are able to inspect the metadata as it is attached as attributes to the R data frame. In our example, we first inspect the metadata of the datasets. This can help in understanding data and guide decisions on merging data into synthesis data products. The R script covering the steps of inspecting the metadata is available via Gists (https://gist.github.com/cpfaff/f0ac88924b5cbde48949). The scripts covering the analysis parts and the visualization can be found here https://gist.github.com/cpfaff/3dc94da4f6191fedaad1 and here https://gist.github.com/cpfaff/63ecba903b4b4b8a4783 (Hothorn et al. 2008, Weisberg 2011, Pinheiro et al. 2014). Furthermore, the script containing all code of our examples in one file is available via figshare http://dx.doi.org/10.6084/m9.figshare.1365364 and Gist from here https://gist.github.com/cpfaff/c7dcfa1c971ee61150b2.

The data of other researchers are only accessible if they made it public or when the access has been granted through a paper proposal. From rBEFdata, the access is controlled via credentials in which a registered user can find on its profile page on a BEFdata instance. The credentials can be set via the command *bef.options(“user_credentials” = “xyz”)*. After the approval of a proposal on BEFdata, it is possible to batch download all requested datasets. The batch download requires the ID of the proposal, which can be found in its URL. The proposal we use in our example contains three datasets and it has the ID 90 (Fig.1). We import the datasets using the command *bef.portal.get.datasets.for_proposal(id = 90)* (Fig.2). We need two of the datasets for our example analysis and we assign these datasets to different variables. We call them “n_retention” and the other “design”. A look into the column headers of these both datasets reveals a common column header named “plot_id”. If we want to use that column to merge the two datasets into a synthesis product, we need to ensure that they contain the same categories and have the same meaning. As rBEFdata provides access to the EML formatted metadata, we have access not only to the title, the abstract but also to the column descriptions of each dataset we just downloaded. We can access the metadata using the R builtin *attributes()* command (e.g., *attributes(n_retention)*). We can check whether the two datasets can be merged by the “plot_id” column inspecting the column descriptions from the metadata. For example, for the dataset we just assigned to the variable “design,” we can inspect the column description issuing the command *attributes(design)$columns[1,]$description*. The description from the first dataset can then be compared with the description of the “plot_id” column in the second dataset (Fig.3).

## Analysis and Postprocessing Documentation

**Figure 4:**
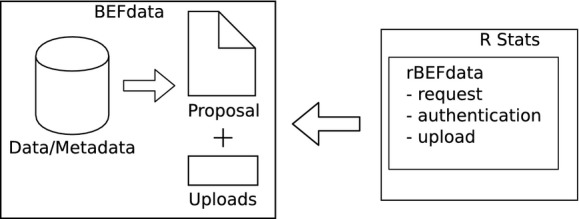
After the analysis has been performed, derived datasets, images, as well as the R script itself can be uploaded and directly attached to the original paper proposal. In our example, we upload a plot in PNG format, depicted in Figure5, and the R script as attachment to the proposal which is shown in Figure S1. Uploading the R script used for analysis allows to keep a whole provenance record linking the primary data with derived products. The R code covering the steps of data upload we provide a Gist on GitHub https://gist.github.com/cpfaff/37c131c7a6b903db2f00. Furthermore, the script containing all code of our examples in one file is available via figshare http://dx.doi.org/10.6084/m9.figshare.1365364 and Gist from here https://gist.github.com/cpfaff/c7dcfa1c971ee61150b2.

**Figure 5:**
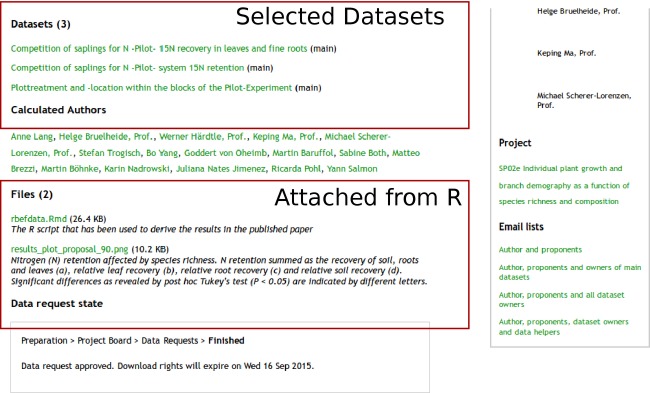
The proposal page shows the primary data put together to answer a certain research question. After completing the analysis, derived data products can be attached in with no limitation to a specific format. In our example, we attach a plot figure and an R script that has been used to derive the results. As we show, it is possible to add a description to the attachment which allows to provide more information. The description for the plot figure we attached here provides similar information like a figure caption on how to interpret the results.

After the import of the data, the data preparation, and the merging of the data into a synthesis data product, we can start with our analysis (Lang et al. 2014; fig. 3). In our example analysis, we create a plot figure representing our results (Figs.3, S1). We attach the R script containing the analysis and the plot figure we created to the proposal for documentation (Fig.4). We can attach to proposals using the command *bef.portal.attach_to_proposal(id = xx, attachment = “path/to/file”, description = “desc”)*. We need to provide the command with the ID of the proposal we want to attach to, a path to a file we want to attach, and a description. Documenting the postprocessing steps in the form of a script and derived data products like a plot figure together with the paper proposal preserves valuable information (Fig.5).

## Discussion

As part of the rOpenSci community (http://ropensci.org/), we aim to make scientific data available from within R that is typically spread across many databases and networks around the world (e.g., DataOne, KNB, GBIF). With rBEFdata, we provide one piece to the puzzle offering convenient access to data and metadata hosted on instances of the BEFdata data management platform (Nadrowski et al. 2013). The core functionality of the rBEFdata package is quite similar to what other packages offer which allow to access scientific data from within R (e.g., rDataOne, rGBIF). By the time of writing, to the best of our knowledge, the rBEFdata package was the first R package persisting EML structured metadata over the import of data into an analysis workflow with R. Furthermore, the support and the access to metadata from R is not yet widely spread (e.g., see rDataOne). However, we currently work on a more generic framework for R that supports read and write of metadata as well as import and export of data based on the EML metadata standard (https://github.com/ropensci/EML). This framework can then be used to provide other R packages with the functionality and the benefits that come along with having access to metadata. Metadata has a wide range of applications during scientific analysis workflows. We highlighted how it can be used to guide the decisions on manually merging primary data into synthesis products. Metadata also represents a first step toward automatic data integration. If the metadata, for example, includes unit information, this can be used to level out granularity differences making the creation of synthesis data products easier. However, a full automatic assemblage and integration of data would need further support of higher-order semantics in the form of ontologies (Michener and Jones 2012).

Although thesauri may be of limited use as direct help in full automatic data integration, they provide important guidance toward that step. They can be employed to structure harmonization efforts within and across projects, providing mechanisms to agree on concepts and definitions which helps with speaking a common language. A controlled vocabulary applied over data can simplify manual data integration and potentially help in linking data to ontologies (Michener and Jones 2012). Furthermore, thesauri have the potential to be used for data discovery as we have shown in our example. With rBEFdata, we start to provide this important kind of functionality based on TemaTres, as the thesaurus of the Long-Term Ecological Research network (LTER) is based on this technology (Porter et al. 2011). Reusing existing vocabulary is attractive as this can save more effort. However, reuse is not always an option if there is no vocabulary available that suits the project. As TemaTres is open source provided under a GPL license, this allows any project to set up an own instance for the development of a project-specific vocabulary (http://www.vocabularyserver.com/). The flexibility to switch between vocabularies in rBEFdata allows projects to create and use a vocabulary on their own or just decide to reuse existing vocabularies of their choice. Searching BEFdata from R, however, currently has some limitations: As data on BEFdata instances are private by default, there is no way to use the respective data right away except it has been made public by the data owner. According to the workflow defined in BEFdata, a proposal has to be written to gain access to the data, but there is currently no functionality implemented that would allow to generate a paper proposal from R. The creation of proposals from R, however, is planned for future releases of rBEFdata which then allows to fully exploit the potential of the search mechanism.

Scientific workflow tools like Kepler or Pegasus have a long tradition in documenting the postprocessing of data (Altintas et al. 2004; Oinn et al. 2004; Deelman et al. 2009). Using the attachment functionality of rBEFdata with the paper proposal mechanism of BEFdata provides a simple way to use documentation mechanism for the postprocessing of data from within R. An extensive use of the attachment functionality significantly increases the value of paper proposals as they can become a single point of reference holding all information necessary to understand and reproduce a scientific analysis. Our documentation mechanism, however, currently is very application-specific to BEFdata. Thus, we are working on combining our documentation mechanism with features based on ideas of reproducible reporting in R (Yihui Xie 2014). BEFdata will then provide export functionality of paper proposals holding all their information which could be published along with primary data and the paper. Although journals today more often require the data products used to derive the results as a prerequisite for publication, the link to the primary data is lost. Without this information, however there is no way to control for underlying errors that might occur on data preparation steps, which typically represent about 70% of the whole scientific analysis workflow (Garijo et al. 2014). Providing the primary data and the postprocessing information together with the publication would allow to control for underlying problems which is important as improperly designed, or incorrectly analyzed experimental data can lead to incorrect conclusions (Tilman 1989). Publishing primary data and postprocessing information along with a paper has the potential to improve the reliability and reproducibility of scientific findings.

## Conclusion

In creating the rBEFdata R package as a companion to the open source data management portal BEFdata (Nadrowski et al. 2013), we provide a convenient tool to communicate with BEFdata servers directly from R. The open source licensing of BEFdata and its companion R package rBEFdata allow research projects to run their own sophisticated data management, curation, analysis, and documentation setup. Combining the R package with the data management platform can significantly improve the data analysis workflow, the productivity, and collaboration in any ecological project while promoting best practices in the data management and reproducibility through good documentation.

## Supporting Information

**Figure S1.** The plot we created to visualize the results in our analysis example. The code covering the steps to create the pot figure is available from here https://gist.github.com/cpfaff/63ecba903b4b4b8a4783. For a detailed explanation of the results see (Lang et al. 2014).
