# Supplementary figures and images for: rBEFdata: documenting data exchange and analysis for a collaborative data management platform
Source: Ecol Evol. 2015 Jul 3;5(14):2890–7. doi: 10.1002/ece3.1547 (PMC4541993; doi:10.1002/ece3.1547)

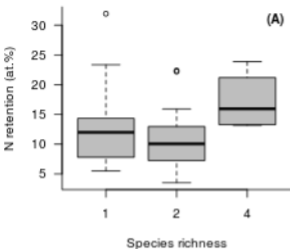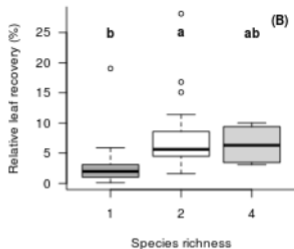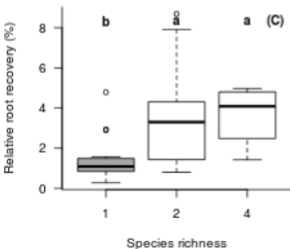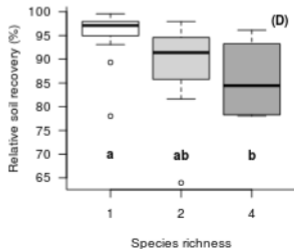

Supplement: Supplementary file 1 [file ece30005-2890-sd1.pdf]
